# State-Dependent Changes in Auditory Sensory Gating in Different Cortical Areas in Rats

**DOI:** 10.1371/journal.pone.0126684

**Published:** 2015-04-30

**Authors:** Renli Qi, Minghong Li, Yuanye Ma, Nanhui Chen

**Affiliations:** 1 School of Life Sciences, University of Science and Technology of China, Hefei, Anhui, P. R. China; 2 State Key Laboratory of Brain and Cognitive Science, Kunming Institute of Zoology, Chinese Academy of Sciences, Kunming, Yunnan, P. R. China; 3 Yunnan University of Traditional Chinese Medicine, Kunming, P. R. China; 4 State Key Laboratory of Brain and Cognitive Science, Institute of Biophysics, Chinese Academy of Sciences, Beijing, P. R. China; 5 Yunnan Key Laboratory of Primate Biomedical Research, Kunming, Yunnan, P.R. China; University of Jyväskylä, FINLAND

## Abstract

Sensory gating is a process in which the brain’s response to a repetitive stimulus is attenuated; it is thought to contribute to information processing by enabling organisms to filter extraneous sensory inputs from the environment. To date, sensory gating has typically been used to determine whether brain function is impaired, such as in individuals with schizophrenia or addiction. In healthy subjects, sensory gating is sensitive to a subject’s behavioral state, such as acute stress and attention. The cortical response to sensory stimulation significantly decreases during sleep; however, information processing continues throughout sleep, and an auditory evoked potential (AEP) can be elicited by sound. It is not known whether sensory gating changes during sleep. Sleep is a non-uniform process in the whole brain with regional differences in neural activities. Thus, another question arises concerning whether sensory gating changes are uniform in different brain areas from waking to sleep. To address these questions, we used the sound stimuli of a Conditioning-testing paradigm to examine sensory gating during waking, rapid eye movement (REM) sleep and Non-REM (NREM) sleep in different cortical areas in rats. We demonstrated the following: 1. Auditory sensory gating was affected by vigilant states in the frontal and parietal areas but not in the occipital areas. 2. Auditory sensory gating decreased in NREM sleep but not REM sleep from waking in the frontal and parietal areas. 3. The decreased sensory gating in the frontal and parietal areas during NREM sleep was the result of a significant increase in the test sound amplitude.

## Introduction

Sensory gating, a process in which the brain’s response to a repetitive stimulus is attenuated, is thought to contribute to information processing by enabling organisms to filter extraneous sensory inputs from the environment. To date, sensory gating has typically been used to determine whether brain function is impaired in response to an environmental stimulus, such as in individuals with schizophrenia or addiction [1–6]. Sensory gating can be adequately assessed using the sound stimuli of a Conditioning-testing paradigm, in which two identical auditory tones are presented 500 ms apart. Normal humans and rats exhibit a smaller response to the second (test) tone compared with the first (conditioning) tone [7, 8]. A positive wave that occurs 50 ms (P50) after the auditory stimuli represents a widely used auditory evoked potential (AEP) component to assess sensory gating in humans. In rats, a negative wave (N40) that occurs 40 ms after the auditory stimuli, which is considered analogous to the P50 recorded in humans, has generally been used [9–11].

In healthy subjects, sensory gating is sensitive to a subject’s behavioral state, such as acute stress or attention [12, 13]. Cortical responses to sensory stimulation are decreased during sleep because the activation of the ascending reticular activating system, which maintains the waking state, decreases, and the inhibitory activities of the thalamocortical system increase [14]. However, information processing continues throughout sleep as evidenced by the fact that a sleeper will reliably be aroused by increasingly intense stimuli or the familiar sound of an alarm clock [15]. Furthermore, researches regarding the effects of a vigilant state on AEPs have demonstrated that an AEP can also be elicited by sound during NREM and REM sleep. For example, Nir and co-workers demonstrated auditory responses and stimulus-specific adaptation in the rat auditory cortex are preserved across NREM and REM sleep [16]. Moreover, the AEP amplitude is significantly higher during NREM sleep compared with both waking and REM sleep [17–20]. However, it is not known whether sensory gating changes during sleep.

Furthermore, electroencephalogram (EEG) [21–23] changes in different cortical areas from waking to sleep states are non-uniform. For example, during NREM sleep, the spectra power maps of healthy human EEGs indicate that the delta band power predominates in the frontal cortex, the theta band power predominates in the occipital cortex, and the alpha band predominates in the parietal cortex [24]. A lower frequency power (delta power) predominates in the frontal cortex during NREM sleep, which suggests the neural activities of this region are less than the neural activities in other cortical areas and may be a consequence of more intensive use in daytime [23, 25]. In accordance with this interpretation, sleep deprivation (40 hours) induced the greatest increase in EEG slow-wave activity at the frontal cortex [21], and positron emission tomography demonstrated that the prefrontal cortex exhibits the largest reduction in activation in whole cortical areas during NREM sleep from waking in healthy humans [26]. The regional differences in neural activities during sleep indicate that sleep is a non-uniform process in the whole brain [23]. Thus, the question arises as to whether sensory gating changes in different cortical areas from waking to sleep states are uniform.

To address these questions, we used the sound stimuli of a Conditioning-testing paradigm to examine auditory sensory gating during waking, NREM and REM sleep states in different brain areas (e.g., the frontal, parietal and occipital areas) in rats.

## Materials and Methods

### 1. Animals

The experiments were performed on seven adult male Sprague-Dawley rats (Animal House Center, Kunming Medical College, China) that weighed 250–350 g (8–10 weeks old). The rats were housed under standard lighting (14:10-hour light:dark cycle, lights on at 7:00 am), humidity (50–60%) and temperature (22–23°C) conditions. Food and water were available ad libitum. The care and treatment of the rats complied with the guidelines for the National Care and Use of Animals approved by the National Animal Research Authority (P.R. China), and the experiments were approved by the Institutional Animal Care and Use Committee (IACUC) of Kunming Institute of Zoology (approval ID SYDW-2012020).

### 2. Surgery

**Fig 1:**
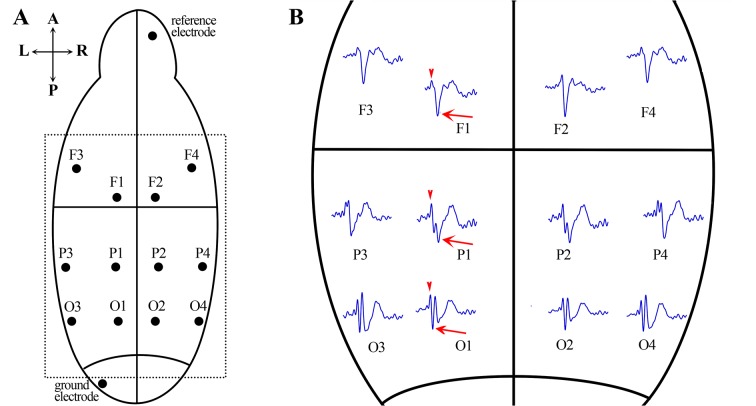
Arrangement of electrodes and AEP waveforms recorded from different cortical areas. **A.** Twelve straw electrodes were symmetrically placed over the frontal, parietal and occipital areas; one electrode was placed over the olfactory area as a reference electrode, and one electrode was placed over the cerebellum as a ground electrode. **B.** An expanded drawing of the area in the dashed box in the left figure. The waveforms represent the responses to the first sound recorded from different cortical areas in a waking rat. The arrows indicate the N1 component peaks, and the arrow heads indicate the peaks of the positive component (P1) before the N1 component.

The EEG electrodes were implanted via surgery. The rats were anesthetized with pentobarbital sodium (50 mg/kg, intraperitoneal injection; Shanghai Chemical Factory, China), and the surgery occurred during deep anesthesia, which was monitored by the toe pinch reflex test. The rats were placed in a rat stereotaxic instrument using blunt ear bars, which protected the middle ear from injury. After a midline scalp incision, fourteen small holes (1.0 mm, diameter) were drilled in the skull according to a rat brain atlas (Paxinos and Watson, 2007). The electrode arrangement is shown in Fig 1, A: four skull screw electrodes (1.2 mm, diameter) were placed over the frontal areas, including **F1** (A–P: 0.5 mm, M–L: 1.0 mm), **F2** (A–P: 0.5 mm, M–R: 1.0 mm), **F3** (A–P: 2.0 mm, M–L: 3.5 mm), and **F4** (A–P: 2.0 mm, M–R: 3.5 mm); two screw electrodes were placed over the parietal areas, including **P1** (A–P: -3.5 mm, M–L: 1.0 mm) and **P2** (A–P: -3.5 mm, M–R: 1.0 mm); two screw electrodes were placed near the temporal areas, including **P3** (A–P: -3.5 mm, M–L: 4.0 mm) and **P4** (A–P: -3.5 mm, M–R: 4.0 mm); four screw electrodes were placed over the occipital areas, including **O1** (A–P: -7.0 mm, M–L: 1.0 mm), **O2** (A–P: -7.0 mm, M–R: 1.0 mm), **O3** (A–P: -7.0 mm, M–L: 4.0 mm), and **O4** (A–P: -7.0 mm, M–R: 4.0 mm); one screw used as the reference electrode was inserted into the skull over the olfactory bulb (A–P: 12.0 mm, M–L: 2.0 mm); and one screw used as the ground electrode was placed on the skull over the cerebellum (A–P: -12.0 mm, M–L: 2.0 mm). All socket connectors (Flexible Flat Cable (FFC) connector, 15 pins, Shenzhen King-hunter Technology Co., Ltd.) connected to the screw electrode were inserted into an electrode pedestal. The electrodes and pedestal were fixed on the skull with dental acrylic cement. Penicillin was administered two days after the surgery via an intramuscular injection (0.2 million units per day). The electrode-implanted rats were gently returned to their home cage and allowed to recover for 2 weeks prior to EEG recording.

### 3. Apparatus for EEG recording

All EEG recordings were performed during the light phase of the light/dark cycle and under freely moving, unrestrained conditions. After surgical recovery, the rats (10–12 weeks old) were allowed to adapt in a sound-proof box (45*45*45 cm), which contained their home cages (30*23*15 cm), for 2 days with white noise (65 dB) and paired-click stimuli (80 dB) for 5 hours (9:00 am to 2:00 pm) per day. A flexible flat cable (15 channels, 1.5 cm width) connected the connectors on the rat’s head to the amplifier. Recording was performed on the third day from 9:00 am to 2:00 pm. The EEG signals from all electrodes were amplified and digitized by a commercial biophysical amplifier (Symtop UEA-FZ 41, Symtop Instrument Co., Ltd), which included a 16-bit A/D, resolution 0.5 μV, input range ± 15 mV, sampling rate 1000 Hz, and band-filtered from 0.1 to 120 Hz, with an analog notch filter at 50 Hz; the data were subsequently saved and displayed on a computer. Rat behavior was monitored by a video camera, which was set approximately 1 m above the rat’s cage; the video data were synchronously saved by a computer. The EEG and behavior recording software were custom-made by Microsoft Visual Studio 8 with a software development kit of the commercial biophysical amplifier (Symtop).

### 4. Stimulus Paradigm

The sound stimuli of the Conditioning-testing paradigm were produced by Psychtoolbox-3 (http://psychtoolbox.org/) in MATLAB (MathWorks, Natick, MA) and delivered to the rat in the recording process. Two identical sound stimuli (5000 Hz, 10 ms duration) were delivered with a 500 ms Inter-Stimulus Interval (ISI) at 80 dB (SPL) and infrequently with a 5 to 10 s random Inter-Trial Interval (ITI) to prevent habituation. The auditory click stimuli were presented with a loudspeaker (E3010, vigoole) mounted approximately 35 cm immediately above the floor of the animal’s cage.

### 5. Data Analysis

The EEG data were analyzed off-line by our custom-made software, which was also programmed by Microsoft Visual Studio 8. First, the data were band-filtered by a digital filter from 1 to 40 Hz. Second, the data were divided into 3 components (waking, NREM sleep and REM sleep) by three individual’s visual examinations. The criteria for the differentiation of the three vigilant states by EEG signals and behavior videos are based on previously published methodology [27]. The waking state is characterized by low-amplitude, fast EEG activity (alpha or beta oscillations) and a substantial number of behavioral activities in the behavior videos. NREM sleep is characterized by high–amplitude, slow EEG activity in a delta range (1–4 Hz) with characteristic waveforms of sleep spindles and K-complexes and still behavior in the videos. In contrast, REM sleep is characterized by short-time duration, low-amplitude (no more than 2 minutes), fast EEG activity (dominated by theta oscillations) and completely still behavior in the videos. The segments of waking, NREM and REM sleep EEG used to analyze the AEP were the common parts of components differentiated by three individuals.

The three states of data were averaged and independently analyzed. First, the AEP signals were separated from the raw data. For each trial, the AEP data collection was initiated 100 ms prior to the stimulus onset and lasted for 400 ms after each stimulus was completed. Second, an AEP waveform was computed by averaging 150 AEP trials. The waveforms in response to the conditioning and test sounds were independently analyzed. We define the negative waveform that occurred at 30–60 ms following the auditory stimuli as the ‘N1’ component (Fig 1B arrows). The waveform amplitudes evoked by the conditioning sound (CAMP) and the test sound (TAMP) were determined as the absolute difference between the peak of the P1 (the positive component before N1, Fig 1B arrowheads) and the N1 component. A ratio of the amplitudes, the TAMP/CAMP or “T/C” ratio, was computed to quantify sensory gating. A T/C ratio close to 0 indicates a robust suppression, whereas a T/C ratio of 1 indicates essentially no sensory gating. Finally, the sensory gating of different brain areas during waking, NREM and REM sleep were statistically analyzed.

All data were subsequently processed with the SPSS (version 13; SPSS, Chicago, IL, USA) software package for statistical analysis. The distribution characters of the AEP waveforms in different cortical areas in the waking state were analyzed first. The differences in the auditory sensory gating, N1 amplitudes of conditioning (CAMP) and test sound (TAMP), and N1 latencies of conditioning (CLAT) and test sound (TLAT) in the right and left cortical areas were tested by the Wilcoxon paired test, for the symmetric arrangement of electrodes (**F1** vs. **F2**, **F3** vs. **F4**, **P1** vs. **P2**, **P3** vs. **P4**, **O1** vs. **O2** and **O3** vs. **O4**). The effects of the cortical area on the auditory sensory gating, CLAT, TLAT, CAMP and TAMP were investigated using one-way repeated-measures ANOVAs, respectively. The signals from the six electrodes on the left cerebral hemisphere were subsequently used to analyze the effects of the vigilant state on auditory sensory gating, CLAT, TLAT, CAMP and TAMP because of the bilateral symmetrical characteristics of the AEP waveforms (Fig 1B). One-way repeated-measures ANOVA for each cortical area was used to examine the effects of the vigilant state on the auditory sensory gating, CLAT, TLAT, CAMP and TAMP, respectively, with the Bonforreni correction post hoc test. Finally, the differences in the increments in the TAMPs and CAMPs from waking to NREM sleep were assessed using one-way repeated-measures ANOVA too. A statistically significant standard was set at *P*<0.05 with a 95% confidence interval.

## Results

### 1. AEP waveforms in different cortical areas in waking rats

The AEP waveforms evoked by the conditioning sound during waking in different cortical areas are shown in Fig 1B. The waveforms in the anterior and posterior areas were very different from each other (e.g., the AEP waveform recorded at **F1** vs. **P1** vs. **O1**). However, visual examination cannot distinguish the AEP waveforms of cortical areas in the left brain from their counterparts in the right brain (e.g., the AEP waveform recorded at **F1** vs. **F2**). The most prominent AEP component in our study was the N1 that occurred at 30–60 ms after the auditory stimuli, which was used to quantify sensory gating.

The detailed N1 latencies (CLAT and TLAT), amplitudes (CAMP and TAMP) and values of sensory gating (T/C) in different cortical areas in waking rats are shown in Table 1. Consistent with our visual examination, there was no significant difference in the N1 latency, N1 amplitude or sensory gating between the paired right and left recording sites (**F1** vs. **F2**, **F3** vs. **F4**, **P1** vs. **P2**, **P3** vs. **P4**, **O1** vs. **O2** and **O3** vs. **O4**, all *P*>0.05, Wilcoxon paired test). A one-way repeated measures ANOVA regarding the CLAT, TLAT, CAMP and TAMP among the different cortical areas identified significant differences in all parameters (*F* = 22.33, 21.68, 17.26 and 9.24, *P* = 0.000, 0.000, 0.000 and 0.001, respectively); However, there was no significant difference in sensory gating (T/C) among all cortical areas (*P* = 0.339, one-way repeated measures ANOVA) (Table 1).

**Table 1:** N1 latencies, amplitudes of conditioning and test sounds and values of sensory gating (TAMP/CAMP) in different cortical areas in waking rats.

Areas	CLAT (ms)*	CAMP (μv) *	TLAT (ms) *	TAMP (μv) *	T/C
**F1**	58.1(7.5)	85.87(10.8)	58.14(10.1)	38.0(6.8)	0.45(1.0)
**F2**	58.3(7.7)	86.99(9.0)	58.43(9.9)	39.1(5.0)	0.45(0.1)
**F3**	56. (5.5)	94.54(14.6)	54.71(6.1)	45.6(12.3)	0.43(0.1)
**F4**	55.7(5.9)	110.3(19.5)	54.43(6.3)	54.8(12.6)	0.4(0.1)
**P1**	53.9(14.7)	77.75(8.3)	51.14(14.4)	33.5(8.8)	0.53(0.1)
**P2**	58.3(9.8)	77.63(8.4)	57.71(11.6)	31.9(10.0)	0.51(0.1)
**P3**	55.3(6.1)	68.25(14.1)	53.57(7.2)	35.0(4.0)	0.47(0.1)
**P4**	53.3(12.5)	68.37(14.6)	52.43(13.5)	35.0(11.0)	0.49(0.1)
**O1**	35.4(5.6)	76.71(10.3)	34.71 (5.4)	35.1(8.5)	0.46(0.1)
**O2**	38.3(8.1)	78.33(13.8)	37.71(6.9)	34.5(8.2)	0.44(0.1)
**O3**	33.3(7.3)	39.36(17.3)	31.71(5.7)	19.4(7.0)	0.57(0.3)
**O4**	34.6(7.5)	38.35(12.3)	34.14(5.8)	20.2(4.9)	0.54(0.1)

There are significant differences in the CLATs, TLATs, CAMPs and TAMPs in different cortical areas, but no differences in auditory sensory gating. The data are expressed as the mean (SD). “*” represents *P*<0.05, one-way repeated measures ANOVA.

### 2. State-dependent changes in auditory sensory gating in different cortical areas

**Fig 2:**
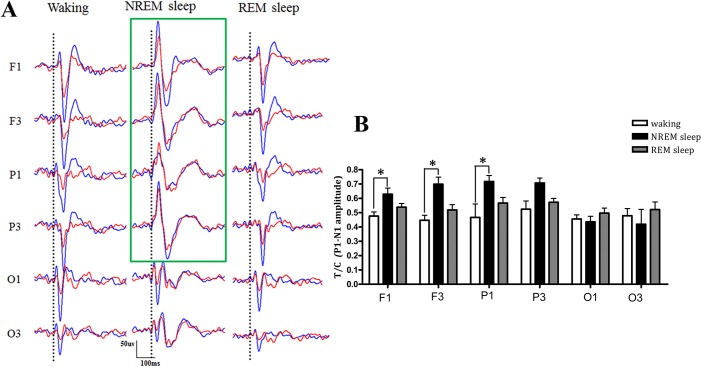
Auditory sensory gating during waking, NREM sleep and REM sleep in different cortical areas. **A.** The AEP waveforms to the Conditioning-testing paradigm sound stimuli during waking, NREM sleep and REM sleep in different cortical areas are shown. Blue and red AEP waveforms were evoked by the conditioning and test sounds, respectively; the dotted line indicates the start of the sound stimuli. The green rectangle indicates the substantial changes in the sensory gating and AEP waveforms in the frontal and parietal areas during NREM sleep. **B.** The T/C values of the left cortical areas during waking, NREM and REM sleep. The data indicate that auditory sensory gating was effected by vigilant states in the frontal and parietal areas (**F1, F3, P1,** and **P3**), but not in the occipital areas (**O1** and **O3**). Moreover, auditory sensory gating recorded in NREM sleep, but not in REM sleep, was decreased (the ratio of T/C increased) from waking in the cortical areas **F1,F3** and **P1**. The data are expressed as the mean ± SEM; “*” represents *P*<0.05, one-way repeated-measures ANOVA, with Bonferroni correction post hoc tests.

As shown in Fig 2A, there were prominent differences in the AEP waveforms evoked by the conditioning and test sounds between waking and NREM sleep, but not between waking and REM sleep, in the frontal and parietal areas (**F1, F3, P1** and **P3**); however, there were no such differences in the occipital areas (**O1** and **O3**). One-way repeated-measures ANOVA regarding each cortical area (**F1, F3, P1, P3, O1** and **O3)** indicated the state had an effect on the T/C values in the frontal and parietal areas (**F1, F3, P1** and **P3**) (*F* = 11.18, 6.01, 8.29 and 6.25, *P* = 0.002, 0.016, 0.005 and 0.014, respectively), but not in the occipital areas (**O1** and **O3**). Post hoc tests that used the Bonferroni correction indicated that the T/C values during NREM sleep, but not during REM sleep, were increased from waking in the cortical areas **F1**, **F3** and **P1** (NREM vs. waking, *P* = 0.017, 0.047 and 0.029, respectively) (Fig 2B).

### 3. State-dependent changes in the N1 latency and amplitude of conditioning and test sounds in different cortical areas

**Fig 3:**
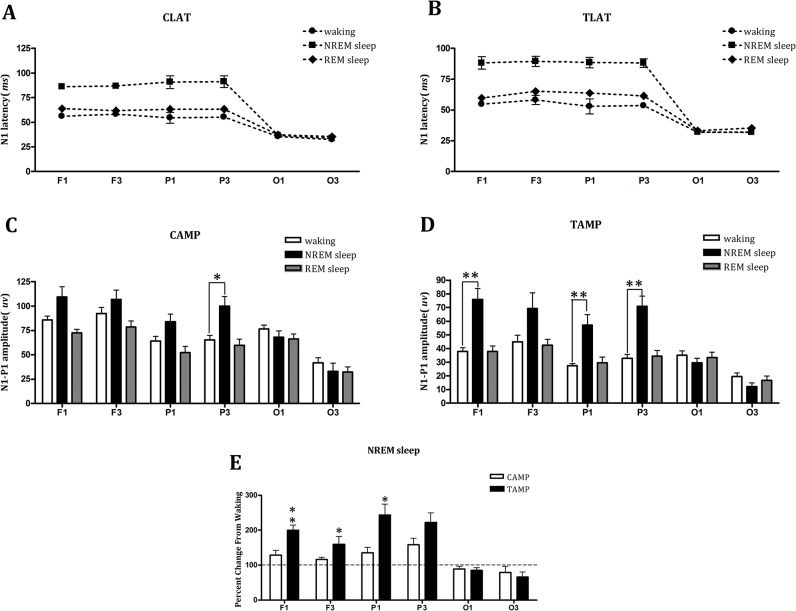
Changes in the N1 latencies and amplitudes of the conditional and test sounds in different cortical areas during different vigilant states. **A,B**. Latencies of the conditional sound (CLATs) (A) and test sound (TLATs) (B) in different cortical areas during waking, NREM sleep and REM sleep. **C,D.** Amplitudes of the conditional sound (CAMPs) and test sound (TAMPs) in different cortical areas during waking, NREM sleep and REM sleep. **E.** The percentages of increments in the TAMPs and CAMPs from waking to NREM sleep in different cortical areas. The data indicate that the CLAT, TLAT, CAMP and TAMP were affected by the vigilant state in the frontal and parietal areas (**F1, F3, P1** and **P3**), but not in the occipital areas (**O1** and **O3**). The data are expressed as the mean ± SEM; “*” represents *P*<0.05, “**” represents *P*<0.01, one-way repeated-measures ANOVA, with Bonferroni correction post hoc tests.

One-way repeated-measures ANOVA for each cortical area (**F1, F3, P1, P3, O1** and **O3**) indicated that the vigilant state had an effect on the CLAT (F = 41.63, 40.76, 21.08, and 7.04, P = 0.000, 0.000, 0.000, and 0.034), TLAT (F = 40.76, 41.68, 21.85, and 75.93, P = 0.000, 0.000, 0.000, and 0.000), CAMP (F = 6.64, 6.05, 8.44, and 8.70, *P* = 0.011, 0.015, 0.005, and 0.005) and TAMP (*F* = 19.38, 5.00, 10.40, and 19.72, *P* = 0.000, 0.026, 0.002, and 0.000) in the frontal and parietal areas (**F1, F3, P1,** and **P3**, respectively), but not in the occipital areas (**O1** and **O3**). Post hoc tests that used the Bonferroni correction indicated that the CLATs and TLATs recorded in NREM sleep were significantly longer from waking in all frontal and parietal areas (**F1, F3, P1,** and **P3**) (CLATs: *P* = 0.002, 0.001, 0.006, and 0.026, respectively; TLATs: *P* = 0.001, 0.002, 0.008, and 0.000, respectively). The CLATs and TLATs recorded in REM sleep were significantly longer from waking in cortical areas **F3** and **P3** (CLAT: *P* = 0.042 and 0.01, respectively; TLAT: *P* = 0.015 and 0.027, respectively) (Fig 3A and 3B). The CAMPs recorded in NREM were significantly larger from waking in the cortical area **P3** (*P* = 0.018) and the TAMPs in the cortical areas **F1, P1,** and **P3** (*P* = 0.003, 0.003, and 0.004, respectively); however, the CAMPs and TAMPs recorded in REM were not significantly different from waking (Fig 3C and 3D). The results demonstrated both the CAMPs and TAMPs in the frontal and parietal cortical areas were increased during NREM sleep from waking. However, further examination of the CAMP and TAMP changes from waking to NREM indicated that the increments in the TAMPs were significantly increased compared with the CAMPs in the cortical areas **F1**, **F3** and **P1** (*P* = 0.003, 0.042 and 0.029, respectively, one-way repeated-measures ANOVA) (Fig 3E), and this result was consistent with the decreased sensory gating in the frontal and parietal cortical areas during NREM sleep as shown in Fig 2B.

## Discussion

In this study, we demonstrated vigilant states have effects on auditory sensory gating in the frontal and parietal areas but not the occipital areas. Auditory sensory gating in NREM sleep was decreased in the frontal and parietal areas from waking. However, auditory sensory gating in REM sleep was not different from waking in cortical areas. The decreased sensory gating (the ratio of T/C increased) during NREM sleep in the frontal and parietal areas resulted from the significant increase in the TAMP.

A substantial number of studies have demonstrated that the AEP amplitude during NREM is larger than waking and REM sleep [17–20], which is consistent with our work. Massimini M and co-workers demonstrate that NREM sleep is characterized by delta (0.1 to 4 Hz) rhythms generated by slow thalamocortical oscillations with approximately a 50% duty cycle; furthermore, in general, cortical cells should spend half their time in a hyperpolarized state and the other half in a depolarized state [28], and AEPs that occurred in a depolarized state during NREM sleep exhibited low amplitude, short latency responses, whereas the other AEPs exhibited high amplitude, long latency responses in a hyperpolarized state [29]. The spontaneous firing rates of cortical neurons during the depolarizing phases of the slow sleep oscillation were as high as during waking and REM sleep [30]; thus, the AEPs in depolarizing phases exhibited a similar N1 amplitude and latency compared with waking and REM sleep. However, when a sound stimulus occurred in the hyperpolarized phase, cortical neurons were activated from their hyperpolarized state, the membrane potential underwent a larger change in voltage than during waking [31], and cortical neurons exhibited prolonged hyperpolarized phases in NREM sleep [29, 31]; thus, the N1 amplitude in NREM sleep was larger than waking and REM sleep. Our results demonstrated that the N1 amplitudes and latencies during NREM sleep in the frontal and parietal areas increased from waking and REM sleep, which is consistent with these previous results.

In previous reports, there have been two reasons for the deficits in sensory gating (the increase in the T/C ratio); one reason is the decrease in the CAMP with no change in the TAMP [32–34], and the other reason is the increase in the TAMP with no change in the CAMP [35–39]. The results in our study are consistent with the second reason because the CAMP and TAMP in the frontal and parietal areas both increased; however, the increment of the TAMP was larger. In the first case, if the CAMP decreased with no changes in the TAMP, the responses to external sounds and the inhibition of a repeat stimulus were reduced. The increased function of the dopamine system, such as systemic amphetamine and cocaine [7] and microinjections of quinpirole in the nucleus accumbens [40], could decrease sensory gating via reduced responses to the conditioning sounds. In the second case, if the TAMP is decreased with no changes in the CAMP, the suppression of the response to a repeat stimulus was reduced without a decrease in the responses to an external stimulus. The decreased function of the cholinergic system could decrease sensory gating via an increased response to the test sounds [32, 33, 41]. Acetylcholine(Ach)-containing neurons of the basal forebrain play an important role in the maintenance of wakefulness, and these neurons discharge during waking, decrease firing during NREM sleep and increase firing during REM sleep [14]. Thus, the decreased auditory sensory gating in the frontal and parietal areas may result from the decreased function of the cholinergic system during NREM sleep.

It is interesting that the state-dependent changes in auditory sensory gating in different cortical areas in our experiment were not uniform. Moreover, the auditory sensory gating, CLAT, TLAT, CAMP and TAMP did not change in the occipital areas during three different vigilant states. However, these parameters in the frontal and parietal areas were significantly different between NREM sleep and waking. One explanation is that the N1 component sources may be different between the cortical areas. The N1 component that occurred 30–60 ms following the sound stimuli, which has been defined as ‘N40’ in many studies [17, 29, 42], was very different regarding the latency between the frontal and occipital areas in the waking rats in our experiment. N1 recorded in the occipital areas, which has a shorter latency, could be generated by the medial geniculate body of the thalamus (MGB) and primary cortical structures within layer 4 [43, 44]. The N1s recorded in the frontal and parietal areas, which have longer latencies, may be contributed by the activities of the hippocampus, the secondary auditory cortex, and the reticular activating system [45]. The effects of sleep on higher structures of the auditory pathway are more obvious than the lower structures [45]; thus, these effects may result in a deficit in sensory gating in the frontal and parietal areas, but no effect in the occipital areas during NREM sleep. Another potential reason for the difference may be the distribution of neurotransmitters related to sensory gating and sleep is non-uniform from the frontal to occipital areas. For example, in recent years, many studies have demonstrated that the deficit in sensory gating was related to decreased function of the cholinergic system [7, 37, 39]. Acetylcholine (Ach) was mainly produced by basal forebrain Ach-containing neurons that discharge during waking, decrease firing during NREM sleep and increase firing during REM sleep [14, 46], and these neurons predominately projected to the frontal cortex [14]. Thus, it is reasonable that auditory sensory gating was deficit in the frontal and parietal areas during NREM sleep but not in the occipital areas. The detailed mechanisms that underlie the differences in sensory gating between different cortical areas in NREM sleep require additional well-designed studies.

In the current study, we demonstrated that sensory gating was decreased in NREM sleep compared with waking, which is different than previous findings. A previous study reported that sensory gating during NREM sleep had no change from waking in rats; however, it was impaired during REM sleep [11]. There are some similarities in our studies; for example, the latencies of the conditioning and test sounds increased during NREM and REM sleep, whereas the amplitude increased during NREM sleep but decreased during REM sleep. The difference in sensory gating may have resulted from the arrangement of electrodes. For example, the reference was placed over the cerebellum in the previous study; however, it was placed over the olfactory bulb in our study. The AEP waveforms were substantially affected by the relative distance between the reference and active electrodes [47]. A different arrangement of active and reference electrodes may alter the morphology or occurrence of peak components [48]. For example, studies that have used a frontal sinus reference (the same place with the olfactory bulb in our work) typically report an AEP waveform in which an N50 or N40 component predominates at the vertex [7, 49, 50]; however, a reference electrode placed in the cerebellar hemisphere contralateral to the active electrode indicates a P40 AEP component predominates at the vertex [47]. Although the N50 was recorded in van Luijtelaar’s work [11], the AEP waveforms reflected the difference between two active electrodes (one electrode in the frontal and another electrode in the parietal-occipital areas). However, a subsequent report regarding the effects of sleep on sensory gating in humans demonstrated that sensory gating was intact during REM sleep [51], which is in accordance with our study.

Taken together, our study was the first study to identify a deficit in auditory sensory gating during NREM sleep in the frontal and parietal areas but not in the occipital areas. The different sources of the N1 component between the cortical areas and the non-uniform distribution of neurotransmitters from the anterior to posterior cortices may underlie the differences in sensory gating in the frontal, parietal and occipital areas.
